# Nobiletin mitigates benign prostatic hyperplasia by suppressing prostate cell proliferation through regulation of cell cycle progression, signaling pathways, transcription factor activity, and the androgen-signaling axis

**DOI:** 10.3389/fphar.2025.1661201

**Published:** 2025-12-08

**Authors:** Jun-Hui Song, Daeun Lee, Byungdoo Hwang, Seon-Kyung Hwang, Sumin Choo, Hyeon Ji Jeong, Hoon Kim, Byungyoon Cha, Seok-Joong Yun, Yung Hyun Choi, Wun-Jae Kim, Sung-Kwon Moon

**Affiliations:** 1 Department of Food and Nutrition, Chung-Ang University, Anseong, Republic of Korea; 2 Institute of Urotech, Cheongju, Chungcheongbuk-do, Republic of Korea; 3 Department of Food and Nutrition, Anyang University, Anyang, Republic of Korea; 4 Pharmacorex Co., Ltd., Daejeon, Republic of Korea; 5 Department of Urology, Chungbuk National University, Cheongju, Republic of Korea; 6 Department of Biochemistry, College of Oriental Medicine, Dongeui University, Busan, Republic of Korea

**Keywords:** benign prostatic hyperplasia, nobiletin, cell cycle, 5α-reductase, NF-κB

## Abstract

**Background and aim:**

Benign prostatic hyperplasia (BPH) involves androgen-driven proliferation with reduced apoptosis. Current 5α-reductase inhibitors can cause adverse effects, motivating safer options. We evaluated whether nobiletin, a polymethoxyflavonoid, mitigates BPH features.

**Methodology:**

*In vitro*, nobiletin was applied to BPH-1 epithelial and WPMY-1 stromal cells to assess anti-proliferative effects. *In vivo* efficacy was tested in a testosterone-induced BPH rat model administered oral nobiletin (1 or 5 mg/kg).

**Results:**

Nobiletin induced G0/G1 phase cell cycle arrest by suppressing cyclin D1, cyclin E, and cyclin-dependent kinase 2 (CDK2), while elevating p21 and p27 expression. Expression of 5α-reductase, androgen receptor (AR), fibroblast growth factor (FGF), epidermal growth factor (EGF), and B-cell lymphoma 2 (Bcl-2) was reduced, whereas Bcl-2–associated X protein (Bax) was increased. Nobiletin modulated phosphorylation of c-Jun N-terminal kinase (JNK) and p38 and suppression of protein kinase B (AKT) phosphorylation. Nobiletin reduced nuclear factor kappa B (NF-κB) DNA-binding activity, which was dependent on JNK and p38. *In vivo*, nobiletin reduced prostate size, weight, and epithelial thickness, accompanied by molecular markers changing in the same direction as *in vitro*. Molecular docking analysis further supported the potential of nobiletin to bind 5α-reductase type 2 at the catalytic site.

**Conclusion:**

These results highlight the potential of nobiletin as a novel therapeutic option for BPH.

## 1 Introduction

Benign prostatic hyperplasia (BPH), a prevalent disorder among men aged 60 years and older, is one of the most frequent symptoms associated with aging (Ye et al., 2024). Hyperplasia of epithelial and stromal compartments enlarges the prostate (Roldán Gallardo and Quintar, 2021). Subsequent urethral compression produces lower urinary tract symptoms, typically weak stream, urgency, and frequency. (Egan, 2016). Testosterone is converted by 5α-reductase to dihydrotestosterone (DHT), and elevated DHT is linked to BPH initiation and progression (Swerdloff et al., 2017; Madersbacher et al., 2019). Finasteride and dutasteride, currently used to treat BPH, are 5α-reductase inhibitors (Hirshburg et al., 2016). Although effective in treating BPH, these drugs can cause sexual adverse effects, including decreased libido, erectile dysfunction, and ejaculatory disorders (Moinpour et al., 2007; Mysore, 2012). Therefore, developing therapies that mitigate these adverse effects is necessary.

DHT activates androgen receptor (AR) signaling that promotes proliferation and differentiation in the prostate (Cai et al., 2011). Growth factors such as fibroblast growth factor (FGF) and epidermal growth factor (EGF) contribute to stromal-epithelial growth in BPH (Khodamoradi et al., 2021). Furthermore, the balance between proliferation and apoptosis is shifted toward proliferation in BPH. Accordingly, regulation of apoptotic signaling is important in BPH, where B-cell lymphoma 2 (Bcl-2) promotes cell survival and Bcl-2–associated X protein (Bax) promotes apoptosis, providing opposing control over epithelial and stromal cell turnover (Pereira Soares et al., 2014). Beyond apoptosis, BPH involves dysregulation of the G1-S checkpoint, which is governed by cyclin D1/cyclin-dependent kinase (CDK) 4 and cyclin E/CDK2 and constrained by the CDK inhibitors p21 and p27 (Karimian et al., 2016). In parallel, proliferative and survival signaling are mediated by the mitogen-activated protein kinase (MAPK) and AKT pathways (Kim and Choi, 2010; Revathidevi and Munirajan, 2019). Based on these features, we assessed whether nobiletin modulates these regulators in our BPH models.

Nobiletin, a flavonoid extracted from the peel of citrus fruits, is recognized for its diverse physiological effects, including anti-inflammatory, antioxidant, and anticancer activities, as well as for the prevention of metabolic syndrome (Goh et al., 2019; He et al., 2016; Li et al., 2014). Previous work showed that nobiletin suppresses prostate cancer cell proliferation (Chen et al., 2014). Given the shared involvement of androgen receptor signaling and 5α-reductase activity in both prostate cancer and BPH (Andriole et al., 2004; Nacusi and Tindall, 2011), we evaluated nobiletin as a candidate for BPH. Here, we estimated the therapeutic potential of nobiletin in BPH using human BPH cell lines (BPH-1 and WPMY-1) alongside a testosterone-induced BPH rat model.

## 2 Materials and methods

### 2.1 Materials

Nobiletin (#N1538) was purchased from Sigma Aldrich (St. Louis, MO, United States). SP600125 (#420119) were obtained from Calbiochem (La Jolla, CA, United States). SB203580 (#BML El286) was obtained from Enzo Life Science (Farmingdale, NY, United States). Supplementary Table S1 contains a summary of all antibodies utilized in this study.

### 2.2 Cell culture

The BPH-1 and WPMY-1 cell lines were obtained from the American Type Culture Collection (ATCC, Manassas, VA, United States) and maintained in RPMI 1640 and DMEM, respectively, supplemented with 10% FBS and 1% penicillin-streptomycin, in a 5% CO_2_ incubator at 37 °C, as previously described (Song et al., 2020).

### 2.3 Cell viability assay

Cell viability was assessed using the 3-(4,5-dimethylthiazol-2-yl)-2,5-diphenyltetrazolium bromide (MTT, #M5655, Sigma Aldrich) assay. BPH-1 cells (3 × 10^3^ cells/well) and WPMY-1 cells (4 × 10^3^ cells/well) were seeded in 96-well plates (#3595, Corning Life Sciences, Tewksbury, MA, United States) with 200 μL of medium and incubated at 37 °C for 24 h. After removing the culture medium, nobiletin (0, 30, 60, and 120 μM) diluted in 0.1% dimethyl sulfoxide (DMSO, Duchefa Biochemie Inc., RV Haarlem, Netherlands)/medium was added to the BPH-1 and WPMY-1 cells for 24 h. Following the removal of the previous medium, 100 μL of fresh medium containing MTT solution (5 mg/mL) was added to each well and incubated for an additional 4 h. After the reaction, the medium was aspirated, and 200 μL of DMSO was added to each well. The plate was incubated for 15 min to dissolve the formazan crystals. After incubation, absorbance was measured at 540 nm using a microplate reader (Thermo Fisher Scientific, Waltham, MA, United States).

### 2.4 Cell counting assay

BPH-1 cells (1 × 10^5^ cells/well) and WPMY-1 cells (1.5 × 10^5^ cells/well) were seeded in 6-well plates (#3516, Corning Life Sciences) with 2 mL of medium and incubated at 37 °C in a humidified atmosphere with 5% CO_2_ for 24 h. After removing the culture medium, nobiletin (0, 30, 60, and 120 μM) diluted in 0.1% DMSO/medium was added to the cells and incubated for an additional 24 h. After removing the supernatant, 0.25% trypsin-EDTA (#11570626, Gibco, Grand Island, NY, United States) was added to detach the cells. The number of cells was counted using a hemocytometer and microscope (Optika, Ponteranica, Italy).

### 2.5 Cell cycle analysis

Treated cells were collected, washed with phosphate-buffered saline (PBS), and fixed in 70% ethanol at −20 °C for 4 h. After fixation, the cells were washed three times with PBS and centrifuged at 2,264 × *g* for 5 min at 4 °C to obtain a cell pellet. RNase A and the DNA-intercalating dye, propidium iodide (PI), were then added, and the cells were incubated for 2 h at room temperature in the dark. Cell cycle distribution was analyzed by flow cytometry using a Muse Cell Analyzer (Luminex, Austin, TX, United States) after propidium iodide staining.

### 2.6 Immunoblot analysis

BPH-1 cells (7 × 10^5^ cells/plate) and WPMY-1 cells (9 × 10^5^ cells/plate) were seeded in 100 mm cell culture dishes (#430167, Corning Life Sciences) and incubated for 24 h at 37 °C. The cells were treated with nobiletin at concentrations of 0, 30, 60, and 120 μM and incubated for another 24 h. After treatment, the cells were washed twice with PBS and lysed with 200 μL of lysis buffer. The cell lysates were vortexed and stored at 4 °C for 10 min. This process was repeated three times. The lysates were then centrifuged at 13,000 × *g* for 10 min at 4 °C, and the supernatant was collected. Protein concentration in the supernatant was determined using the BCA protein assay kit (#A55860, Thermo Fisher Scientific), and equal amounts of protein (30 μg/well) were loaded onto a sodium dodecyl sulfate polyacrylamide gel for electrophoresis (SDS-PAGE). Following electrophoresis, the separated proteins were transferred onto nitrocellulose membranes (#10600002, Cytiva, Marlborough, MA, United States). The membranes were washed with Tris-buffered saline with Tween 20 (TBS-T; 20 mM Tris [pH 7.5], 150 mM NaCl, 0.1% Tween 20) and blocked with 5% bovine serum albumin (BSA) in TBS-T for 2 h at room temperature. After blocking, the membranes were washed with TBS-T and incubated overnight at 4 °C with a primary antibody diluted 1:1,000 in TBS-T containing 2.5% BSA. The following day, the membranes were washed with TBS-T and incubated with a secondary antibody diluted 1:5,000 in TBS-T containing 2.5% BSA for incubated for 2 h at room temperature. The membranes were then washed with TBS-T and visualized using the SuperSignal West Pico PLUS Chemiluminescent Substrate reagent kit (#34580, Thermo Fisher Scientific) and the WSE-6200 LuminoGraph II system (ATTO, Tokyo, Japan). Protein bands were quantified using the ImageJ program (National Institutes of Health, Bethesda, MD, United States).

### 2.7 Electrophoretic mobility shift assay

BPH-1 cells (7 × 10^5^ cells/plate) and WPMY-1 cells (9 × 10^5^ cells/plate) were seeded in 100 mm cell culture dishes (#430167, Corning Life Sciences) and incubated for 24 h at 37 °C. The cells were treated with nobiletin at concentrations of 0, 30, 60, and 120 μM and incubated for another 24 h. For inhibitor experiments, cells were pretreated with SB203580 (15 μM) or SP600125 (15 μM) for 1 h and nobiletin 120 μM was added without removing the inhibitor for the remaining 24 h. After treatment, cells were washed twice with PBS and lysed using a lysis buffer. The lysates were incubated on ice for 15 min, after which 20 μL of 10% Nonidet P-40 (NP-40) was added. The samples were centrifuged at 13,000 × *g* for 5 min at 4 °C to separate the cytoplasmic and nuclear fractions. After removing the supernatant, the nuclear pellet was re-suspended in an extraction buffer and incubated for 15 min at 4 °C. The samples were centrifuged at 13,000 × *g* for 5 min at 4 °C, and the nuclear extracts were collected. Protein concentration in the nuclear extracts was quantified using the BCA protein assay kit (#A55860, Thermo Fisher Scientific). The oligonucleotide sequences used for the EMSA included a consensus sequence for NF-κB (AGT​TGA​GGG​GAC​TTT​CCC​AGG​C). The NF-κB oligonucleotide was labeled with [γ-32P] ATP using T4 polynucleotide kinase (#M41.03, Promega Corporation, Madison, WI, United States). Nuclear extracts were incubated with the labeled probe in a binding buffer at room temperature for 20 min. The DNA-protein complexes were separated by electrophoresis on a 6% native polyacrylamide gel in 0.5× TBE buffer at 4 °C. The gel was dried using a gel dryer (Model 583 Gel Dryers, Bio-Rad, California, United States) and visualized by autoradiography with an X-ray film at −70 °C overnight.

### 2.8 Testosterone-induced BPH rat model

All animal experiments were conducted in accordance with the guidelines approved by the Institutional Animal Care and Use Committee of Chung-Ang University (A2021029). Male standard deviation (SD) rats, 6 weeks old (body weight average 160 g), were obtained from DBL (Eumseong, Korea) and acclimatized for 1 week in an animal chamber. The chamber was maintained under controlled environmental conditions (20 °C–26 °C, 30%–75% humidity, 12-h light/dark cycle, 150–300 lux, and 10–15 air changes per hour). To eliminate the influence of endogenous testosterone, rats were castrated under inhalational anesthesia with isoflurane (induction 3% in oxygen; maintenance 1.5%–2.0%; oxygen flow ∼1 L/min). BPH was induced by weekly intraperitoneal injections of testosterone enanthate (TE; 6 mg/kg; mibe GmbH Arzneimittel, Brehna, Germany) for 28 days. During the TE exposure period (days 1–28), nobiletin (1 or 5 mg/kg, once daily) was administered by oral gavage. On injection days, the daily nobiletin dose was given prior to the weekly intraperitoneal TE injection. Finasteride (6 mg/kg; Proscar, Merck & Co., Inc., Rahway, NJ, United States) was used as a positive control. Vehicle control groups matched the solvents used for the test articles (Tween 80 for nobiletin; water for finasteride). Body weight was measured weekly throughout the experiment.

### 2.9 Prostate index

At study termination, rats were euthanized in a dedicated chamber by gradual-fill CO_2_ according to the IACUC-approved protocol. The whole prostate was immediately dissected, trimmed of fat and connective tissue, blotted dry, and weighed to obtain the wet weight. The prostate index was calculated as prostate weight (mg) ÷ body weight (g).

### 2.10 Hematoxylin & eosin staining and histological analysis

H&E staining was outsourced to Woodang Network (Chuncheon, Korea). Prostate tissue samples were fixed in 10% neutral buffered formalin, embedded in paraffin, and sectioned into 4 μm-thick slices. The sections were stained with hematoxylin and eosin according to standard protocols. The stained slides were provided by the company, and histological evaluation was performed using a light microscope. Epithelial thickness was quantified on calibrated images in ImageJ as the perpendicular distance from the basement membrane to the luminal surface. For each animal, 10 glands across 3 non-overlapping fields were measured after excluding folded or tangentially cut areas.

### 2.11 Ligand and protein preparation

The crystal structure of human steroid 5α-reductase type 2 (SRD5A2) complexed with oxidized nicotinamide adenine dinucleotide phosphate (NADP^+^; PDB ID: 7BW1) was obtained from the RCSB Protein Data Bank. Protein preparation involved the removal of all water molecules and non-essential ligands, while retaining NADP^+^ as a cofactor to preserve the native catalytic environment (Supplementary Figure S1A). After structure cleaning, missing hydrogen atoms were added and Kollman charges were assigned using AutoDock Tools (v1.5.7). The 3D structure of nobiletin (CID: 72344) was obtained from the PubChem database in SDF format and converted to PDBQT format using Open Babel (v3.1.1) and AutoDock Tools for docking simulations.

### 2.12 Molecular docking

Molecular docking was performed using AutoDock Vina (v1.2.7). The docking grid was centered near the NADP^+^ binding site, with its center coordinates set to X = −24, Y = 13, and Z = 34. The grid box dimensions were defined as 40 × 32 × 32 along the X, Y, and Z-axes, respectively, using a grid spacing of 0.375 Å. The exhaustiveness parameter was set to 32 to ensure thorough sampling of potential binding poses. Docking results were visualized using Discovery Studio Visualizer (v25.1).

### 2.13 Statistical analysis

Data are expressed as mean ± SD unless stated. Group differences were tested by one-way ANOVA with Tukey’s HSD. When variances were unequal, Welch’s ANOVA with the Games–Howell test was used. Assumptions were checked with the Shapiro-Wilk and Levene tests. For control–treatment comparisons, unpaired two-tailed Student’s t-tests were used with Welch correction when needed. All tests were two-sided, and p < 0.05 was considered significant. Analyses were performed in R (stats, rstatix, and multcompView).

## 3 Results

### 3.1 Nobiletin inhibits the proliferation of BPH cells

**FIGURE 1:**
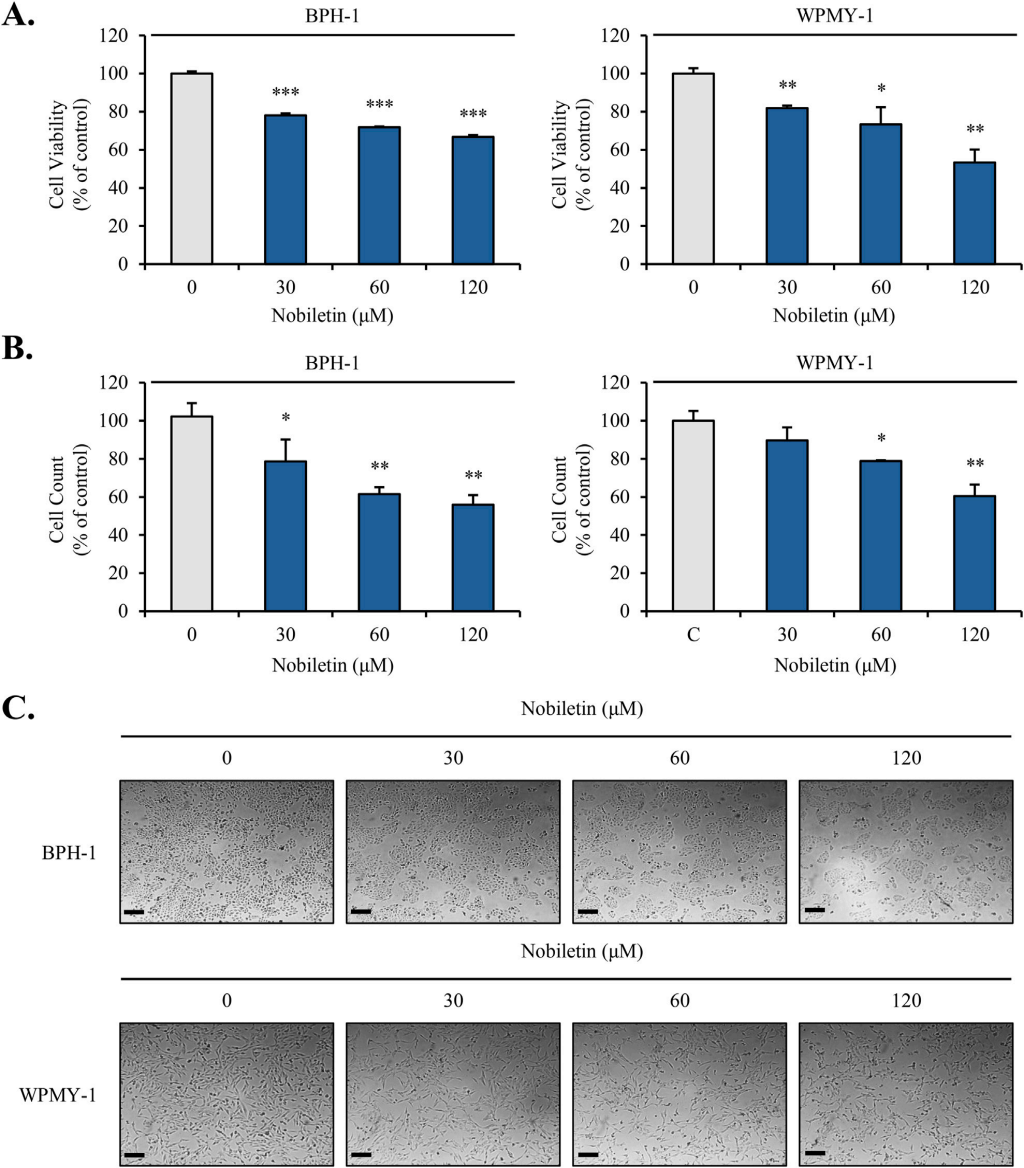
Nobiletin inhibits benign prostatic hyperplasia (BPH) cell viability. BPH-1 and WPMY-1 cells were treated with nobiletin (0, 30, 60, and 120 μM) for 24 h. **(A)** Cell viability was measured by MTT assay. **(B)** Cell numbers were counted using a hemocytometer and presented as fold changes. **(C)** Morphological changes were observed under an inverted microscope (200×). The scale bar represents 50 µm. Data represent mean ± standard deviation (SD) of three independent experiments. *p < 0.05; **p < 0.01; ***p < 0.001.

To evaluate the anti-proliferative effects of nobiletin, viability of the cells was analyzed using the MTT assay. As shown in Figure 1A, nobiletin reduced the viability of both BPH cell lines in a dose-dependent manner. At the highest nobiletin concentration (120 μM), the viability of both BPH cell lines declined by approximately 50% compared to that in the untreated control group. Consistent with the MTT assay results, the cell counting assay revealed a significant reduction in cell numbers following nobiletin treatment (Figure 1B). Furthermore, morphological changes and cell density were observed under an inverted microscope (Figure 1C). These findings demonstrate that nobiletin effectively suppresses the proliferation of both cell lines, with the IC_50_ value estimated to be approximately 120 μM for both cell types.

### 3.2 Nobiletin induces cell cycle arrest of G0/G1-phase in BPH cells

**FIGURE 2:**
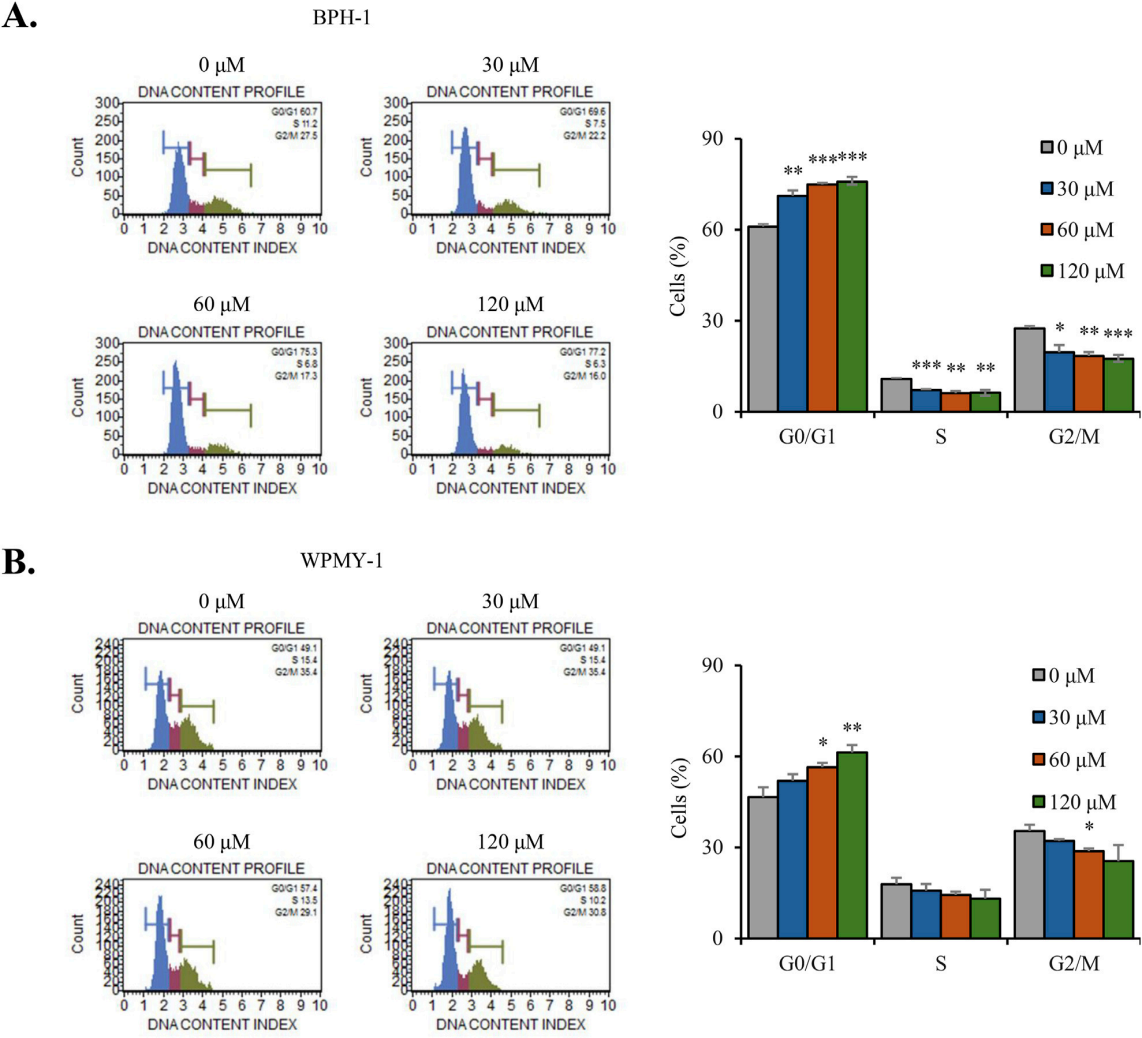
Nobiletin induces G0/G1-phase cell cycle arrest in BPH cells. BPH-1 and WPMY-1 cells were treated with nobiletin (0, 30, 60, and 120 μM) for 24 h **(A,B)** Cell cycle distribution was determined by flow cytometry for BPH-1 and WPMY-1 cells. Bar graphs represent the percentage of cells in each phase. Data represent mean ± SD of three independent experiments. *p < 0.05; **p < 0.01; ***p < 0.001.

We next analyzed the distribution of the cell cycle through flow cytometry. Flow cytometric analysis revealed that nobiletin treatment increased the percentage of cells in the G0/G1 phase, whereas it decreased the population of cells in the S and G2/M phases in both cell lines (Figures 2A,B). These findings indicate that nobiletin treatment inhibits the proliferation of BPH-1 and WPMY-1 cells by inducing G0/G1-phase cell cycle arrest.

### 3.3 Nobiletin regulates the expression of cyclins, CDKs, and cell cycle inhibitors to induce G0/G1-phase arrest in BPH cells

**FIGURE 3:**
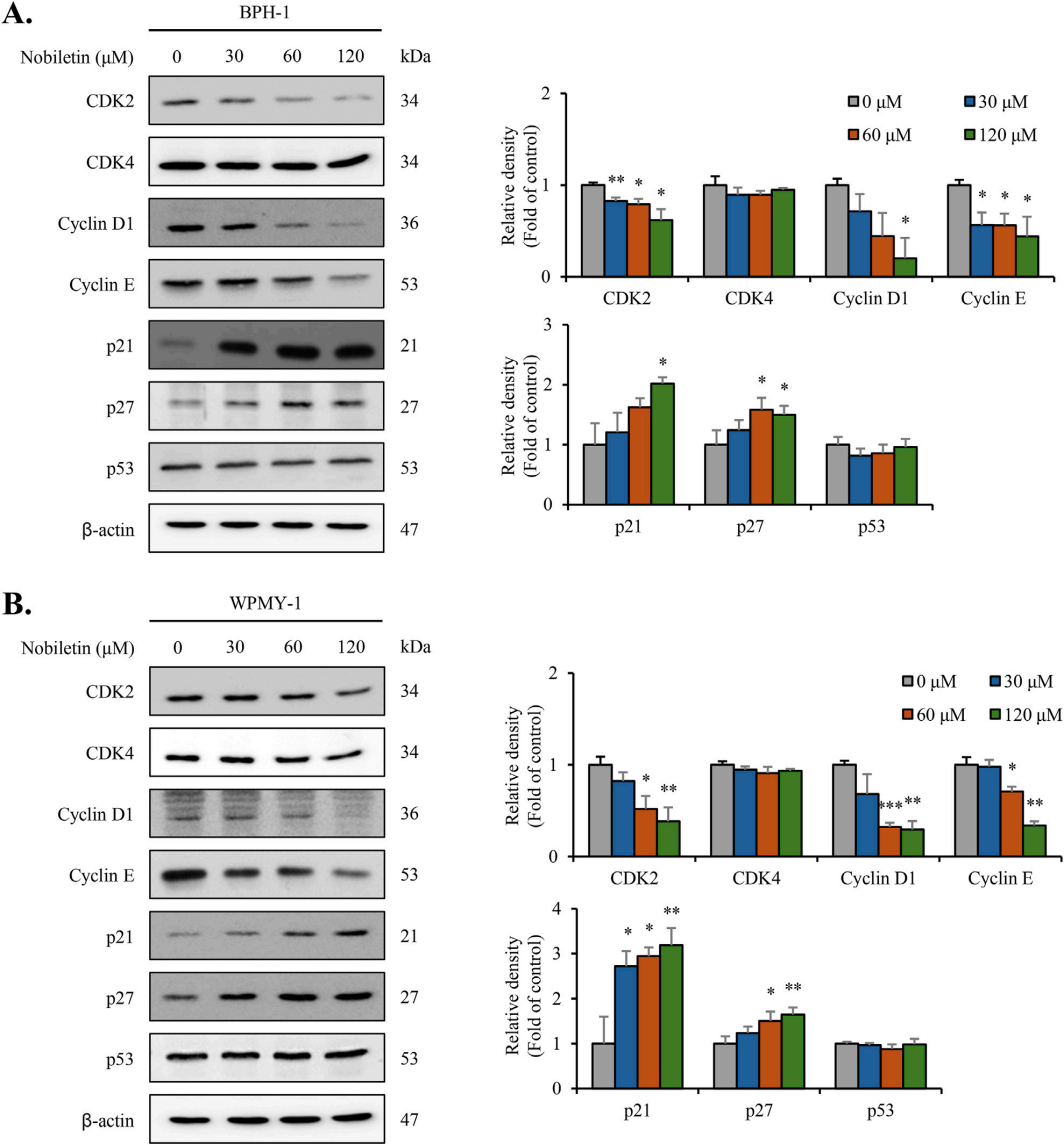
Nobiletin regulates G0/G1-phase cell cycle proteins in BPH cells. BPH-1 and WPMY-1 cells were treated with nobiletin (0, 30, 60, and 120 μM) for 24 h. Expression of cell cycle-related proteins was analyzed by immunoblotting. **(A,B)** Representative immunoblots showing indicated protein levels in BPH-1 and WPMY-1 cells. Protein expression was quantified using ImageJ and normalized to β-actin. Data represent mean ± SD of three independent experiments. *p < 0.05; **p < 0.01; ***p < 0.001.

To clarify the molecular mechanism underlying G0/G1-phase cell cycle arrest caused by nobiletin, immunoblot analysis was performed to assess cell cycle regulatory proteins. Nobiletin treatment significantly reduced the levels of CDK2, cyclin D1, and cyclin E in both BPH-1 and WPMY-1 cells, whereas CDK4 levels remained unchanged (Figures 3A,B). Additionally, the levels of the cell cycle inhibitors p21 and p27 were upregulated following nobiletin treatment, however p53 level showed no significant change in either cell line (Figures 3A,B). These findings suggest that nobiletin induces G0/G1-phase cell cycle arrest in the 2 cell lines by modulating the levels of cyclins and CDKs and by upregulating p21 and p27 cell cycle inhibitors.

### 3.4 Nobiletin regulates the phosphorylation of MAPKs and AKT signaling in BPH cells

**FIGURE 4:**
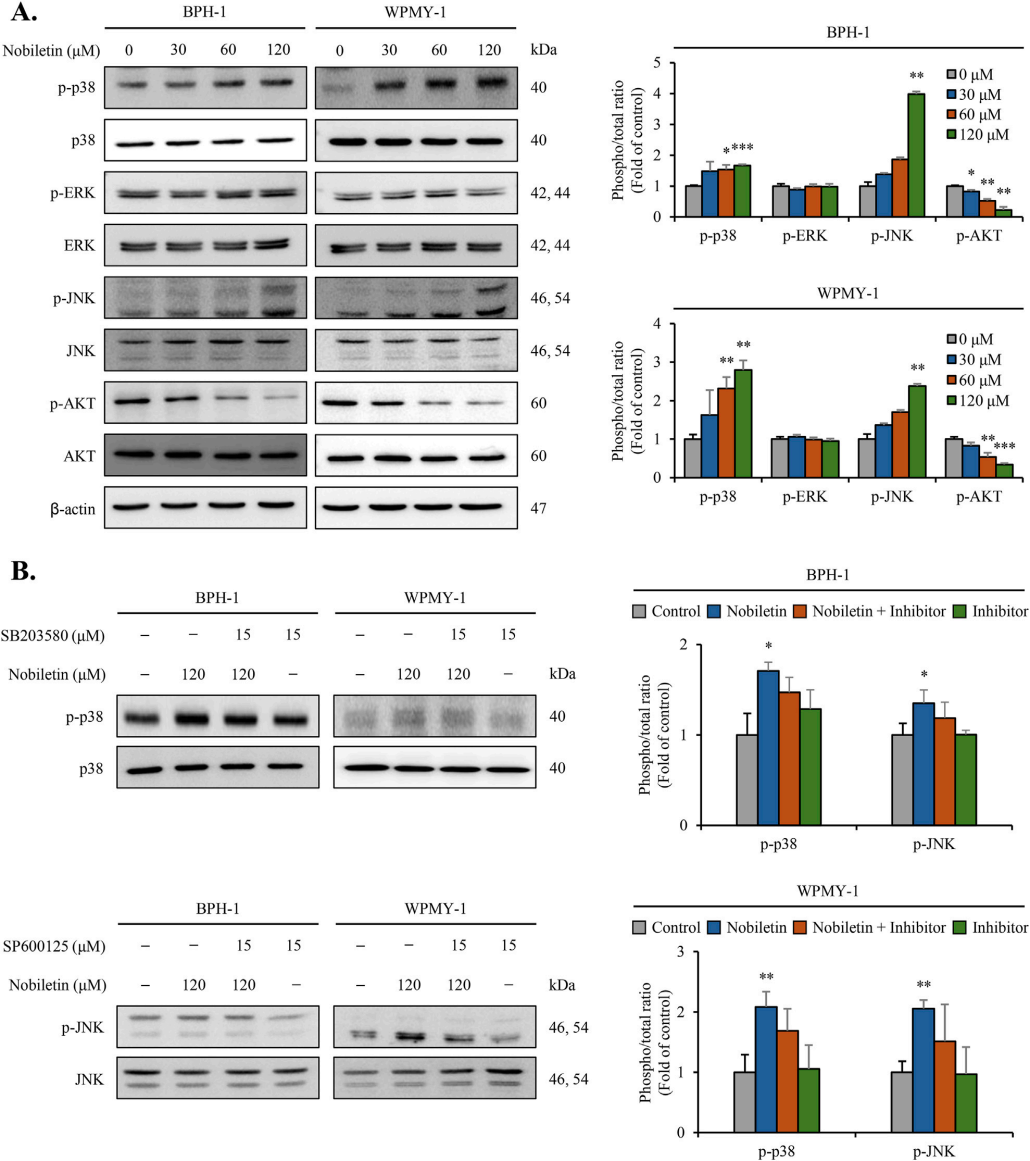
Nobiletin modulates MAPK and AKT phosphorylation in BPH cells. BPH-1 and WPMY-1 cells were treated with nobiletin (0, 30, 60, and 120 μM) for 24 h. **(A)** Total and phosphorylated MAPKs (p38, ERK, JNK) and AKT were analyzed by immunoblotting. **(B)** The phosphorylation levels of p38 and JNK were examined in cells treated with 120 μM nobiletin for 24 h in the presence or absence of their specific inhibitors SB203580 (15 μM, p38 inhibitor) and SP600125 (15 μM, JNK inhibitor). β-actin was used as the housekeeping protein. Quantification of phosphorylated protein levels relative to that of their total forms was performed using the ImageJ software. All data are presented as mean ± standard deviation (SD) from three independent experiments. *p < 0.05; **p < 0.01; ***p < 0.001.

Immunoblot analysis was performed to assess the total and phosphorylated protein levels of ERK, JNK, p38, and AKT signaling molecules. As shown in Figure 4, nobiletin treatment increased the phosphorylation levels of p38 and JNK in a dose-dependent manner in both cells (Figure 4A). Conversely, levels of phosphorylated ERK showed no significant change, whereas those of AKT decreased dose-dependently in both cell lines. Nobiletin-induced phosphorylation of p38 and JNK was remarkably reduced upon inhibitor treatment in both cell lines (Figure 4B). These results demonstrate that phosphorylation of the p38 and JNK pathways is critical for the regulation of cellular signaling by nobiletin.

### 3.5 Nobiletin inhibits NF-κB binding activity in BPH cells

**FIGURE 5:**
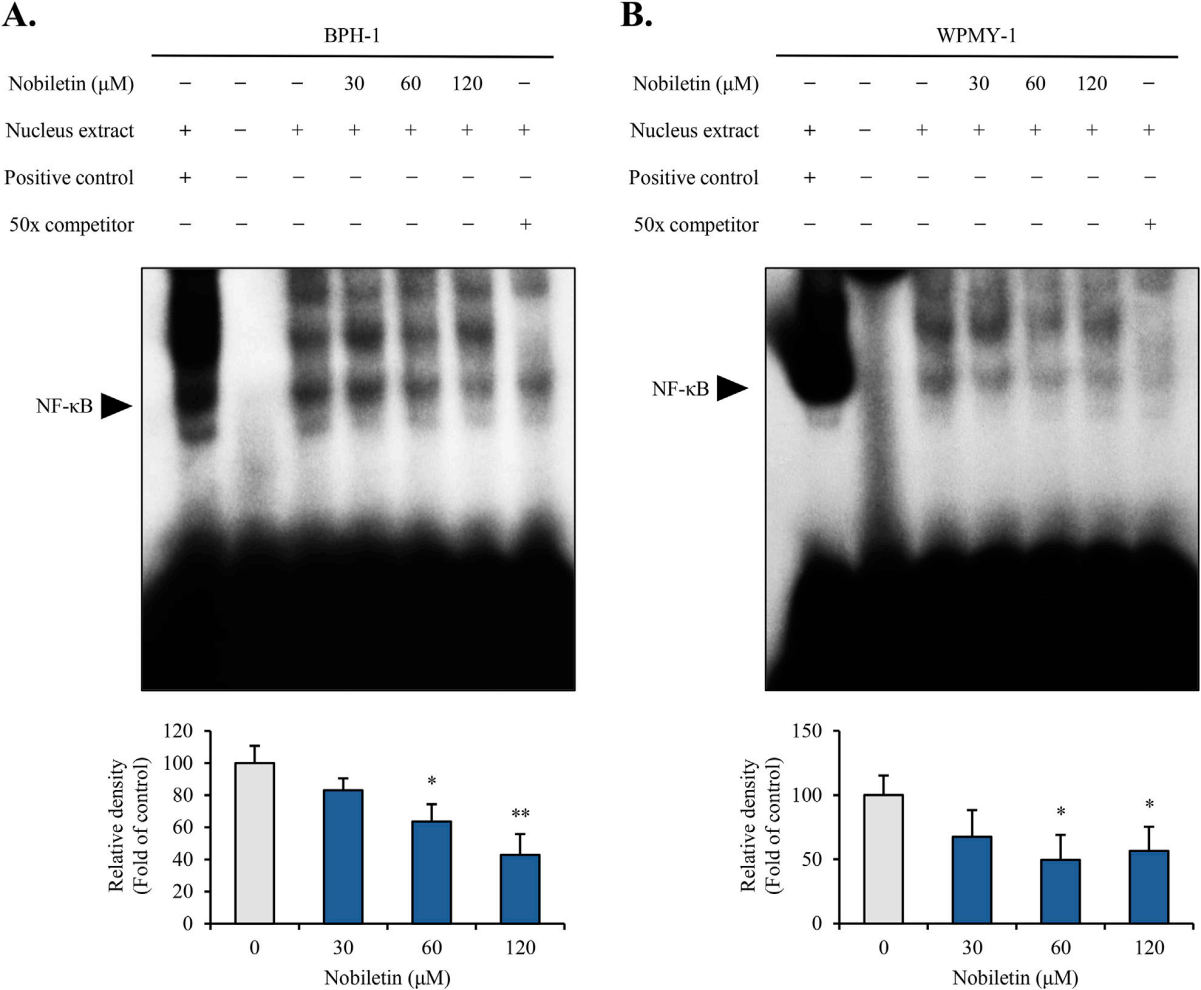
Nobiletin inhibits NF-κB binding activity in BPH cells. BPH-1 and WPMY-1 cells were treated with nobiletin (0, 30, 60, and 120 μM) for 24 h. Nuclear extracts were prepared, and EMSA was performed to analyze NF-κB binding activity. As a positive control, HeLa cell extracts were used, and high concentration (50×) of unlabeled nucleotides for a competitor control were used, respectively. **(A,B)** EMSA images showing NF-κB binding activity in BPH-1 and WPMY-1 cells. All data are presented as mean ± standard deviation (SD) from three independent experiments. *p < 0.05; **p < 0.01.

EMSA was conducted to assess whether nobiletin affects NF-κB DNA-binding activity. The EMSA results showed that nobiletin treatment dose-dependently reduced binding activity of NF-κB in both cells (Figures 5A,B). Addition of JNK inhibitor (SP600125) and p38 inhibitor (SB203580) during nobiletin exposure significantly reversed the reduction in NF-κB binding activity (Supplementary Figure S2A,B). Collectively, these data indicate that nobiletin suppresses NF-κB activity via regulation of JNK and p38 in both BPH cells.

### 3.6 Nobiletin modulates the expression of BPH-associated protein markers in BPH cells

**FIGURE 6:**
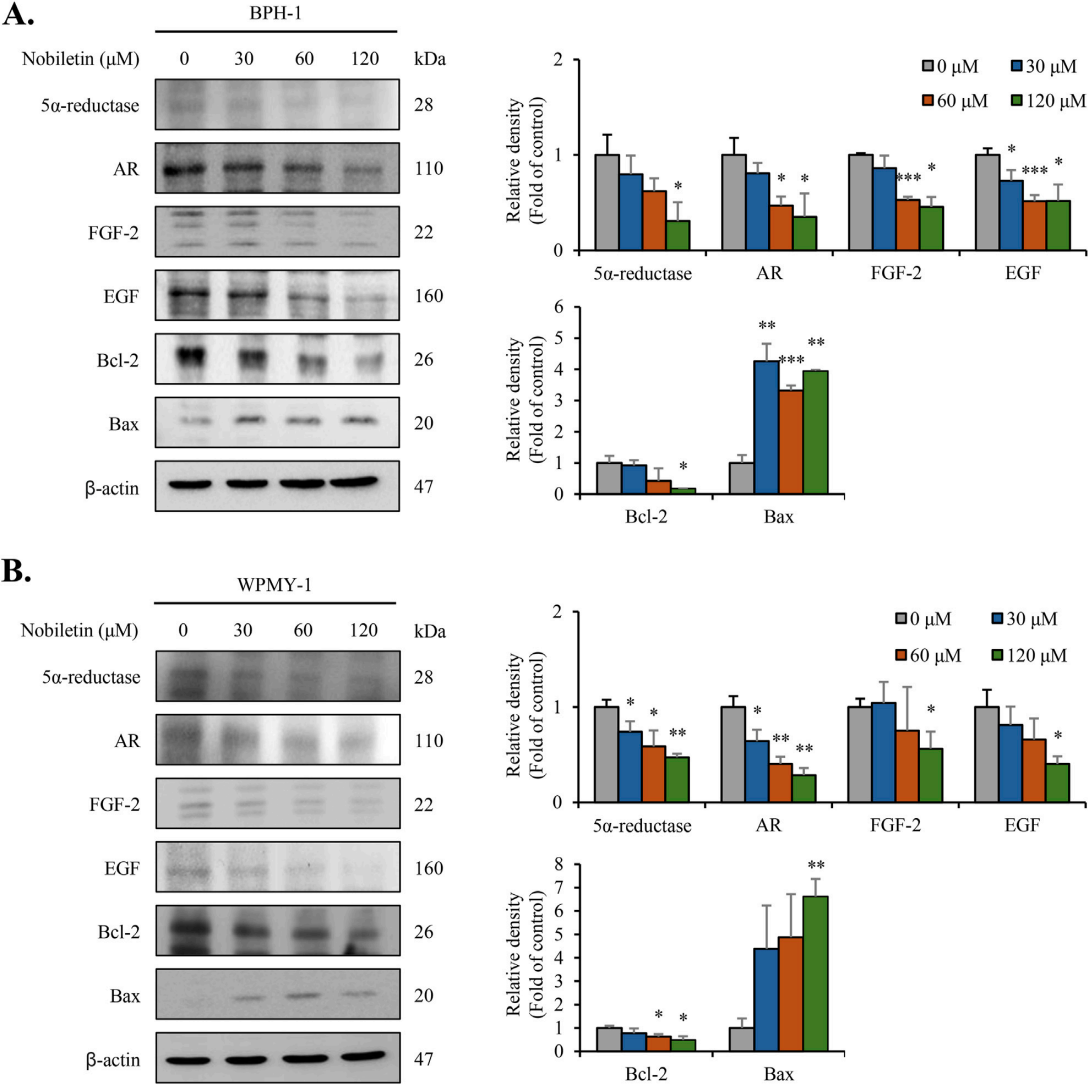
Nobiletin modulates the expression of key BPH-associated protein markers in BPH cells. BPH-1 and WPMY-1 cells were treated with various concentrations of nobiletin (0, 30, 60, and 120 μM) for 24 h and levels of BPH-associated markers, including 5α-reductase, AR, FGF, EGF, Bcl-2, and Bax, were analyzed using immunoblot analysis. **(A,B)** Levels of BPH-associated markers were analyzed using immunoblotting. Quantification of protein expression was performed using ImageJ software, and data are presented as relative expression normalized to that of β-actin. All data are presented as mean ± standard deviation (SD) from three independent experiments. *p < 0.05; **p < 0.01; ***p < 0.001.

To estimate the effects of nobiletin on BPH-associated protein markers, the levels of 5α-reductase, FGF, AR, EGF, Bax, and Bcl-2 were measured in WPMY-1 and BPH-1 cells. Immunoblot analysis revealed that nobiletin treatment dose-dependently decreased the levels of 5α-reductase, AR, FGF, EGF, and the anti-apoptotic protein Bcl-2 in both cell types. Conversely, the expression of Bax was markedly elevated in both the nobiletin-treated cell lines (Figures 6A,B). These results indicate that nobiletin regulates the expression of key BPH-associated markers, potentially contributing to its therapeutic effect on human BPH cell proliferation and viability.

### 3.7 Nobiletin alleviates testosterone-induced BPH in a rat model

**FIGURE 7:**
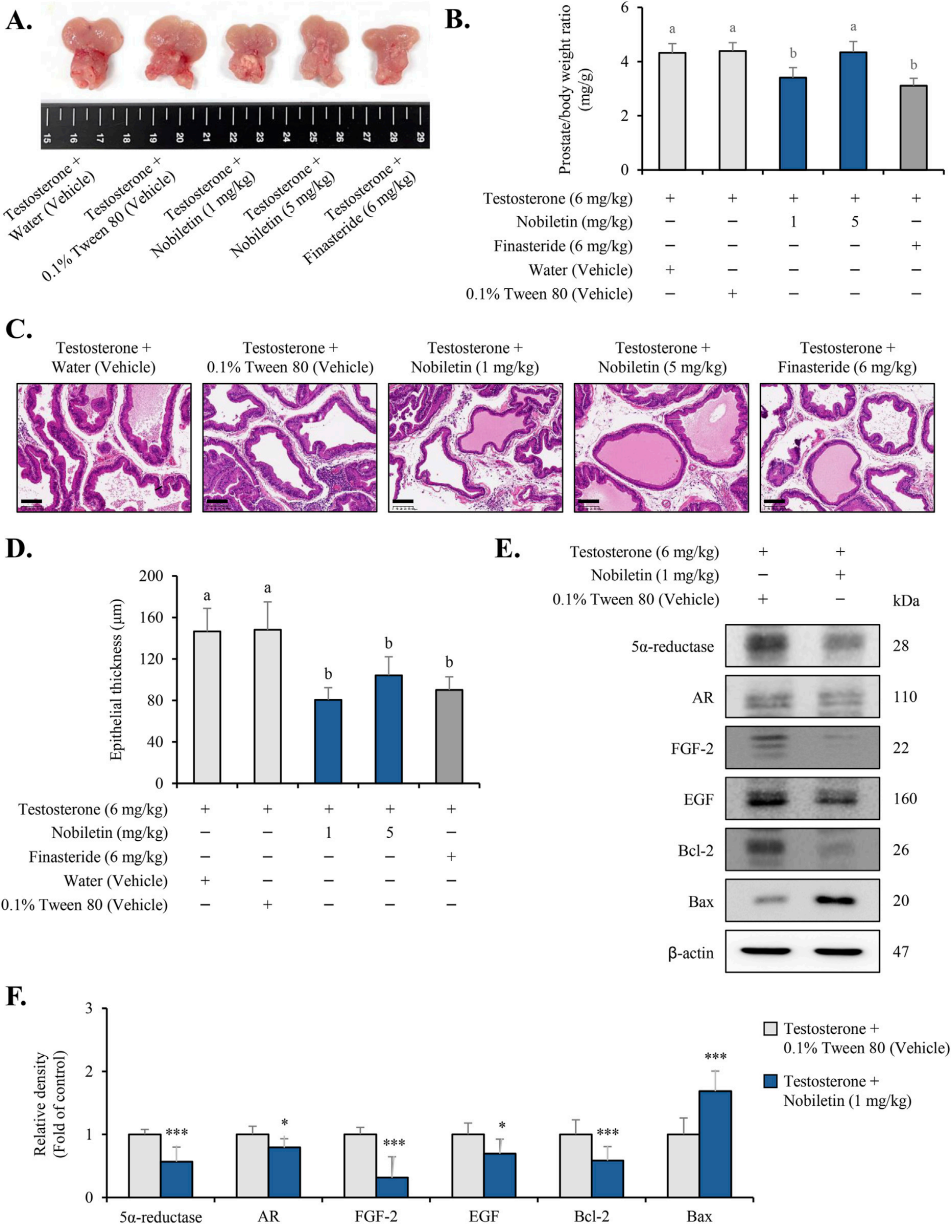
Nobiletin alleviates testosterone-induced BPH in rats. Rats were divided into five groups: water (vehicle), 0.1% Tween 80 (vehicle), nobiletin (1 and 5 mg/kg), and finasteride (6 mg/kg). **(A)** Representative images of prostates from each group showing the macroscopic appearance of the gland after treatment. **(B)** Prostate weight-to-body weight (PW/BW) ratio was calculated to evaluate the effect of nobiletin on prostatic hyperplasia. **(C)** Hematoxylin and eosin (H&E) staining of prostate tissue sections showing the thickness of epithelial and stromal layers in the different treatment groups. The scale bar represents 100 µm. **(D)** Quantitative estimation of the prostatic epithelium thickness in the tissue samples. Nobiletin-treated groups were compared to the 0.1% Tween 80 vehicle group, whereas the finasteride-treated group was compared to the water vehicle group. Statistics **(B,D)** one-way ANOVA across all groups followed by Tukey’s HSD for multiple comparisons. Bars that do not share a letter differ significantly at p < 0.05. **(E,F)** Immunoblotting results of the key BPH markers in prostate tissues. β-actin was used as an internal control. Data are presented as mean ± standard deviation (SD) for each experimental group (n = 7). *p < 0.05 and ***p < 0.001, compared to the 0.1% Tween 80 group.

Testosterone-induced BPH model was used to evaluate the therapeutic efficacy of nobiletin. Macroscopic examination of the prostate revealed that testosterone treatment caused significant prostate enlargement in both the vehicle groups (Figure 7A). However, the prostate size was visibly reduced in rats treated with nobiletin, with the 1 mg/kg nobiletin group showing a reduction comparable to that in the finasteride group. The efficiency of action of nobiletin on prostatic hyperplasia was quantified by assessing the ratio of prostate weight (PW) to body weight (BW) (Figure 7B). Compared to the vehicles, nobiletin treatment (1 mg/kg) markedly diminished the PW/BW ratio. Histological analysis using H&E staining further supported these findings (Figures 7C,D). The vehicle groups displayed prostatic hyperplasia as evidenced by increased thickening of the epithelial and muscle layers. Treatment with nobiletin (1 mg/kg) markedly alleviated these effects and reduced epithelial and stromal hyperplasia to levels comparable to those in the finasteride group (Figures 7C,D). Finally, the levels of key BPH-associated markers (5α-reductase, AR, FGF, EGF, Bcl-2, and Bax) were confirmed in animal study (Figures 7E,F). These results suggest that nobiletin effectively mitigates testosterone-induced BPH with an efficacy similar to that of finasteride.

### 3.8 Nobiletin binds to the catalytic pocket of 5α-reductase type 2

**FIGURE 8:**
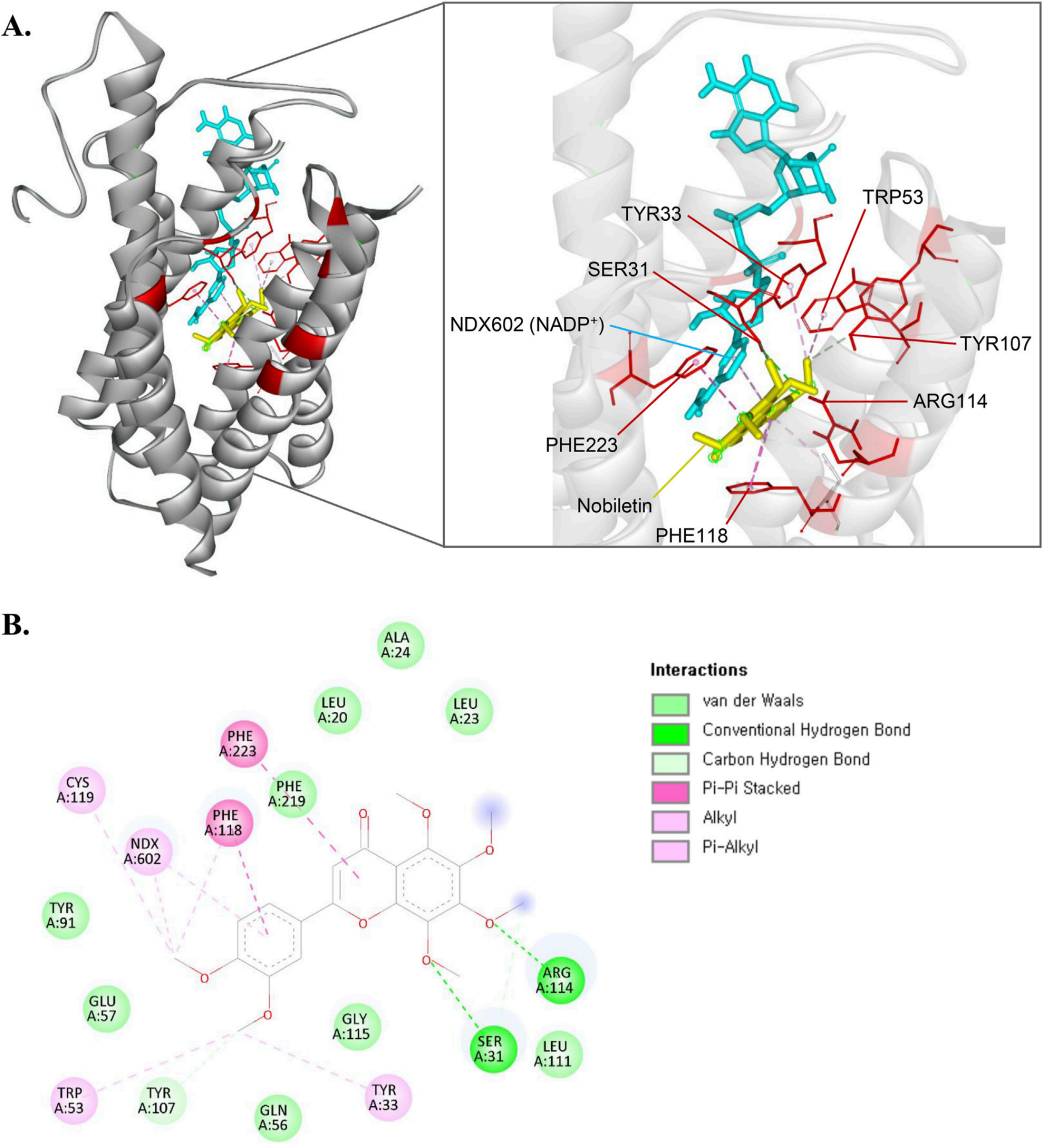
Nobiletin binds within the catalytic pocket of SRD5A2. **(A)** Docking pose of nobiletin (yellow) near the NADP^+^ binding site in the SRD5A2 structure. Key interacting residues, including SER31, ARG114, PHE118, and PHE223, are labeled. **(B)** 2D interaction map showing hydrogen bonds, π-π stacking, and van der Waals interactions between nobiletin and surrounding residues.

To investigate the direct molecular interaction between nobiletin and SRD5A2, molecular docking simulations were conducted with the NADP^+^-bound crystal structure of SRD5A2 (Supplementary Figure S1A). The top-ranked binding pose of nobiletin exhibited a binding affinity of −8.13 kcal/mol (Table1). The docking pose revealed that nobiletin was positioned near the NADP^+^ binding pocket and was surrounded by catalytically important residues (Figures 8A,B). Two conventional hydrogen bonds were formed with SER31 and ARG114, both located at the entrance of the substrate-binding tunnel (Figures 8A,B). Additionally, π-π stacking interactions with PHE118 and PHE223 contributed to stable aromatic interactions within the active site (Figures 8A,B). Hydrophobic and van der Waals contacts with residues such as SER31, TYR33, TRP53 and TYR107 further contributed to ligand stabilization (Figures 8A,B). These findings provide structural insights into how nobiletin may act as a potential inhibitor of 5α-reductase by targeting key catalytic and NADP^+^-adjacent residues.

**TABLE 1:** Binding free energy of nobiletin with 5α-reductase type 2.

Mode	Affinity (kcal/mol)	Root-mean-square deviation	
		Lower bound	Upper bound
1	−8.13	0	0
2	−7.278	1.415	3.396
3	−7.261	2.079	2.882
4	−7.141	1.92	2.321
5	−6.874	2.011	2.711
6	−6.717	1.801	3.474
7	−6.411	2.074	3.168
8	−6.3	8.316	11.09
9	−6.146	2.376	3.327

## 4 Discussion

The findings of this study highlight the potential of nobiletin as a novel therapeutic approach for BPH by targeting key cellular and molecular mechanisms. Using human BPH cell lines (BPH-1 epithelial and WPMY-1 stromal) and a testosterone-induced rat model of BPH, we demonstrated that nobiletin significantly suppresses cell proliferation, causes cell cycle arrest, and modulates the pathways central to BPH progression. These findings offer novel insights and extend previous research on the effects of nobiletin in prostate cancer models (Guney Eskiler et al., 2019). Thus, nobiletin holds promise for BPH management by potentially addressing the imbalance between cell proliferation and apoptosis, which is a key factor in BPH development.

The results of the cell counting and MTT assays confirmed that nobiletin markedly suppressed the proliferation of both human BPH cells with an IC_50_ value of approximately 120 μM. The anti-proliferative effect of nobiletin was further confirmed by its ability to stimulate the arrest of G0/G1-phase cell cycle, as evidenced by flow cytometric analysis. Mechanistically, nobiletin decreased CDK2, cyclin E, and cyclin D1 while increased p21 and p27 in both cell lines, whereas CDK4 and p53 were unchanged. These findings are consistent with prior reports that nobiletin induces G1 arrest by upregulating p21/p27 and suppressing the SKP2–CDK2 axis on renal cell carcinoma (Chen et al., 2021). Evidence in prostate-derived cells further shows increased p21 and G0/G1 arrest with nobiletin (Guney Eskiler et al., 2019). Taken together, these findings indicate that nobiletin exerts anti-proliferative effects by restraining the G1–S transition predominantly through the cyclin E/CDK2–p21/p27 axis in these BPH models.

To further elucidate the role of nobiletin in BPH pathogenesis, we examined its effect on MAPK and AKT signaling pathways, which are the key pathways in mammalian cell communication. The MAPK signaling pathway plays a vital role in modulating diverse cellular functions such as growth, differentiation, metabolism, and apoptosis (Kim and Choi, 2010). The MAPK family comprises ERK, p38, and JNK, all of which play distinct roles in cellular signaling (Kim and Choi, 2010). ERK is predominantly involved in promoting cell proliferation, whereas p38 and JNK are commonly correlated with stress responses, including apoptosis (Kim and Choi, 2010). Similarly, the AKT pathway is critical for cell cycle regulation, growth, metabolism, and cell survival, and often counteracts apoptotic signals and promotes cellular proliferation (Revathidevi and Munirajan, 2019). Our results showed that nobiletin-stimulated activation of p38 and JNK may contribute to its anti-proliferative effects through the induction of stress-related apoptotic pathways. These observations are consistent with prior work showing that nobiletin regulates p38/JNK signaling and impacts downstream stress responses in primary cells (Huang et al., 2016). Additionally, AKT phosphorylation, which is closely associated with cell survival and proliferation, was significantly downregulated following nobiletin treatment, indicating its potential to suppress pro-survival signaling in both human BPH cell lines.

In this study, we also examined a transcription factor NF-κB, which is known to serve as a key regulator of the cell cycle, cell proliferation, inflammation, and apoptosis (Taniguchi and Karin, 2018). NF-κB typically remains in an inactive state in the cytoplasm, where it is bound to the inhibitory protein IκB (Mulero et al., 2019). Upon stimulation by inflammatory cytokines or oxidative stress, IκB undergoes phosphorylation and subsequent degradation, allowing NF-κB to move into the nucleus and control the expression of genes associated with inflammation, survival, and proliferation (Mulero et al., 2019). EMSA results demonstrated that nobiletin markedly hindered the DNA-binding capacity of NF-κB in both cell lines. Moreover, the decrease in NF-κB DNA-binding with nobiletin was reversed by JNK and p38 inhibitors, indicating dependence on these JNK/p38 pathways. Our findings are consistent with studies showing that nobiletin inhibits NF-κB DNA-binding in RAW264.7 cells (Choi et al., 2007) and reduces nuclear translocation of NF-κB in prostate cells (Chen et al., 2014). In addition, nobiletin modulates p38 and JNK with accompanying suppression of NF-κB signaling (Huang et al., 2016). These findings indicate that nobiletin may suppress human BPH cell proliferation through inhibition of NF-κB by regulating JNK/p38 pathway.

BPH-associated protein markers, including 5α-reductase, AR, FGF, EGF, Bcl-2, and Bax, are critically participated in the regulation of prostate growth and apoptosis in BPH (La Vignera et al., 2016; Cardillo et al., 1997). AR and 5α-reductase are critical for androgen signaling, whereas FGF and EGF drive stromal and epithelial proliferation (Azzouni et al., 2012). Our results indicate that nobiletin alleviates BPH progression by regulating the equilibrium between pro-survival and pro-apoptotic factors, thereby reestablishing the balance between apoptosis and cell proliferation. 5α-reductase plays a pivotal role in converting testosterone to DHT, which is directly implicated in prostate enlargement. The observed downregulation of 5α-reductase suggests that nobiletin may reduce DHT levels, thereby alleviating BPH symptoms (Ang et al., 2017). Additionally, the inhibition of AR, FGF, and EGF expression, which are key regulators of stromal and epithelial cell proliferation, highlights the ability of nobiletin to target androgen-driven signaling pathways in prostate cells (Zhu and Kyprianou, 2008). Furthermore, the ability of nobiletin to regulate Bax and Bcl-2, markers that are central to the control of apoptosis, underscores its potential to restore apoptotic balance in BPH-affected cells (Ang et al., 2017). The concurrent decrease of the Bcl-2 and increase of the Bax strongly suggests that nobiletin promotes apoptosis in prostate cells, counteracting the hyperproliferative environment characteristic of BPH.

Nobiletin reduced prostate enlargement and modulated key BPH markers in testosterone-induced BPH rat models, showing efficacy comparable to finasteride. We evaluated low oral doses of 1 and 5 mg/kg, and efficacy was greatest at 1 mg/kg, which by body-surface-area scaling corresponds to 0.16 mg/kg in humans (10 mg/day for a 60 kg adult). The top *in vitro* concentration, 120 μM (∼48 μg/mL), exceeds the reported plasma exposures after oral dosing in rodents (Cmax 3–5 μM at 50 mg/kg) (Wang et al., 2020). Nevertheless, reports of bioactive demethylated metabolites and the *in vivo* effect at 1 mg/kg together support pharmacological plausibility despite the *in vitro-in vivo* gap (Pang et al., 2023). Overall, these data support nobiletin as a potential alternative or adjunctive option for BPH (Nakajima et al., 2020). Although this study focused on key pathways and markers, broader omics approaches may help clarify its mechanism of action. Additional work should also assess its effects in combination with existing therapies and in other BPH models.

Recent structural and computational analyses of SRD5A2 have clarified its substrate recognition and catalytic mechanism through the NADP^+^-bound structure, which revealed how finasteride forms a covalent intermediate with NADP^+^ via hydride transfer and enolization chemistry (Xiao et al., 2020). In our study, nobiletin was predicted to bind near the NADP^+^-binding site, engaging non-covalently with catalytically relevant residues such as ARG114, SER31, and PHE118. These residues have also been implicated in ligand recognition for finasteride (Khantham et al., 2021; Xiao et al., 2020). The binding affinity of nobiletin (−8.13 kcal/mol) was lower than that of finasteride (−10.13 kcal/mol) (Khantham et al., 2021). Because finasteride forms a mechanism-based covalent intermediate with NADP^+^ that is not captured by noncovalent docking, we do not treat score differences as indicators of potency in this system. Recent studies emphasized the importance of PHE118 as a universal hotspot across several ligands, contributing −4.5 kcal/mol for finasteride and up to −9 kcal/mol for α-tocopherol through π-π stacking (Khantham et al., 2021; Xiao et al., 2020). Our results similarly showed that nobiletin forms aromatic interactions with PHE118 and PHE223, suggesting that it replicates the key non-covalent contacts observed in finasteride binding (Khantham et al., 2021; Xiao et al., 2020). Furthermore, the hydrogen bonds formed between nobiletin and ARG114/SER31 resemble the interaction network observed in both finasteride and NADP-dihydrofinasteride (Khantham et al., 2021; Xiao et al., 2020). Although nobiletin showed a lower docking score than finasteride, the predicted contacts with catalytically relevant residues are consistent with plausible noncovalent engagement of SRD5A2. We regard these models as hypothesis-generating and do not infer enzymatic inhibition or binding mode from docking alone. Direct activity assay would be required to establish catalytic inhibition.

In conclusion, our findings demonstrate that nobiletin exerts multi-faceted therapeutic effects against BPH by targeting cell cycle modulation, signaling pathways, and control of NF-κB regulation process (Supplementary Figure S3). These results provide a strong foundation for additional preclinical and clinical studies to explore the potential of nobiletin as a novel therapeutic drug for BPH.

## Data Availability

The original contributions presented in the study are included in the article/Supplementary Material, further inquiries can be directed to the corresponding author.
