# Supplementary material for: Nobiletin mitigates benign prostatic hyperplasia by suppressing prostate cell proliferation through regulation of cell cycle progression, signaling pathways, transcription factor activity, and the androgen-signaling axis
Source: Front Pharmacol. 2025 Dec 8;16:1661201. doi: 10.3389/fphar.2025.1661201 (PMC12722845; doi:10.3389/fphar.2025.1661201)
Supplement: Supplementary file 1 [file Table1.docx]

**Supplementary Data**

| **Table S1. List of antibodies used in this study** | | |
| --- | --- | --- |
| Antibody | Catalogue No. | Working dilution |
| Monoclonal antibody against AR | Santa Cruz (sc-6248) | 1:1000 |
| Monoclonal antibody against FGF-2 | Santa Cruz (sc-1360) | 1:1000 |
| Monoclonal antibody against EGF | Santa Cruz (sc-166779) | 1:1000 |
| Monoclonal antibody against CDK2 | Santa Cruz (sc-6248) | 1:1000 |
| Monoclonal antibody against CDK4 | Santa Cruz (sc-23896) | 1:1000 |
| Monoclonal antibody against cyclin D1 | Santa Cruz (sc-8396) | 1:500 |
| Monoclonal antibody against cyclin E | Santa Cruz (sc-248) | 1:1000 |
| Monoclonal antibody against p27KIP1 | Santa Cruz (sc-1641) | 1:1000 |
| Monoclonal antibody against p53 | Santa Cruz (sc-126) | 1:1000 |
| Monoclonal antibody against β-actin | Santa Cruz (sc-47778) | 1:4000 |
| Monoclonal antibody against p21WAF1 | BD Biosciences (556430) | 1:1000 |
| Polyclonal antibody against phospho-ERK | Cell Signaling (#9101S) | 1:1000 |
| Polyclonal antibody against ERK | Cell Signaling (#9102S) | 1:2000 |
| Polyclonal antibody against phospho-JNK | Cell Signaling (#9251S) | 1:1000 |
| Polyclonal antibody against JNK | Cell Signaling (#9258S) | 1:2000 |
| Polyclonal antibody against phospho-p38 | Cell Signaling (#9211S) | 1:1000 |
| Polyclonal antibody against p38 | Cell Signaling (#9212S) | 1:2000 |
| Polyclonal antibody against phospho-AKT | Cell Signaling (#9271S) | 1:1000 |
| Polyclonal antibody against AKT | Cell Signaling (#9272S) | 1:2000 |
| Secondary antibody against goat anti-mouse | Cell Signaling (#7076S) | 1:5000 |
| Secondary antibody against goat anti-rabbit | Cell Signaling (#7074S) | 1:5000 |
