# Supplementary figures and images for: Nobiletin mitigates benign prostatic hyperplasia by suppressing prostate cell proliferation through regulation of cell cycle progression, signaling pathways, transcription factor activity, and the androgen-signaling axis
Source: Front Pharmacol. 2025 Dec 8;16:1661201. doi: 10.3389/fphar.2025.1661201 (PMC12722845; doi:10.3389/fphar.2025.1661201)

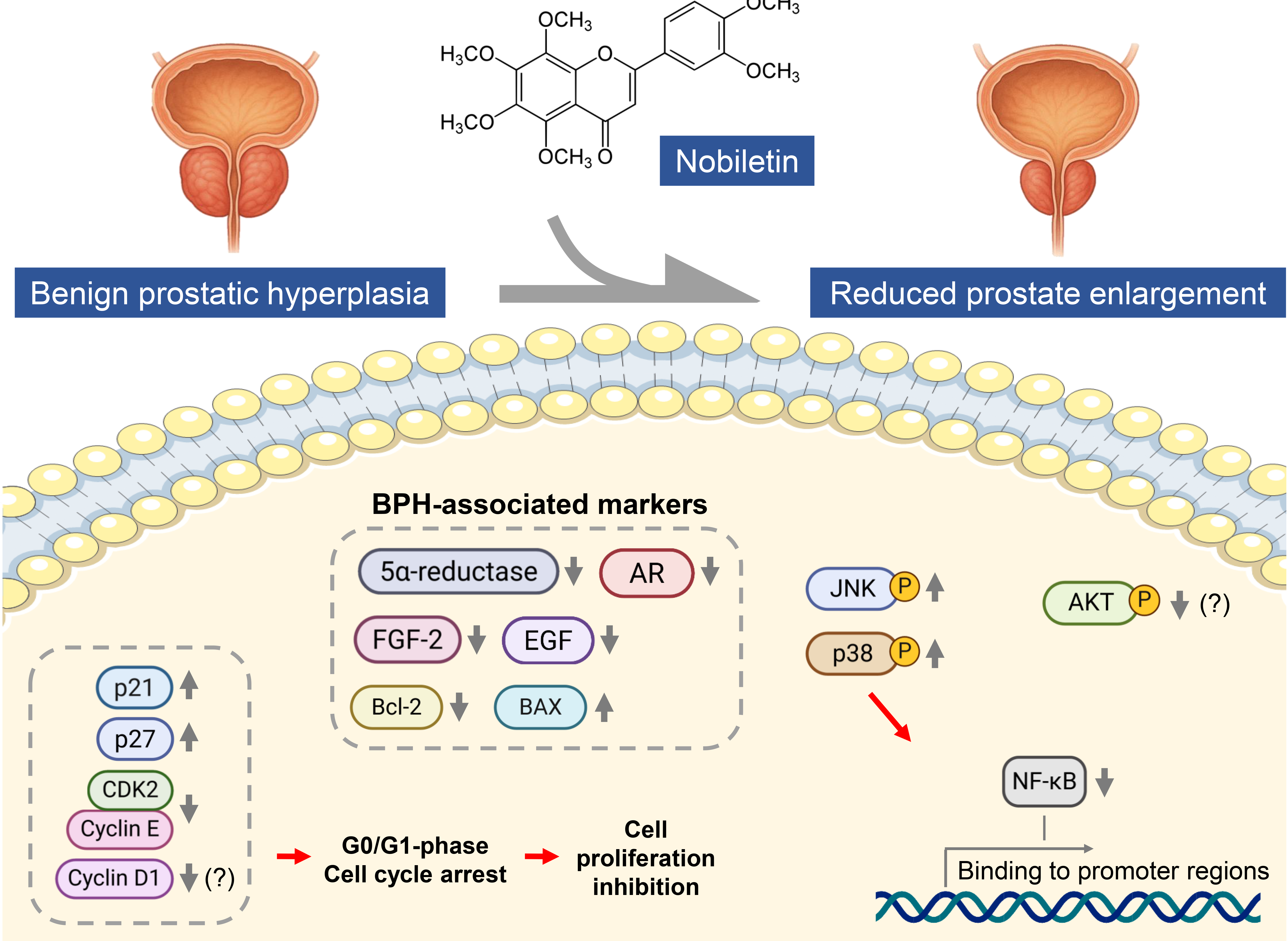

Supplement: Supplementary file 2 [file Image3.tif]

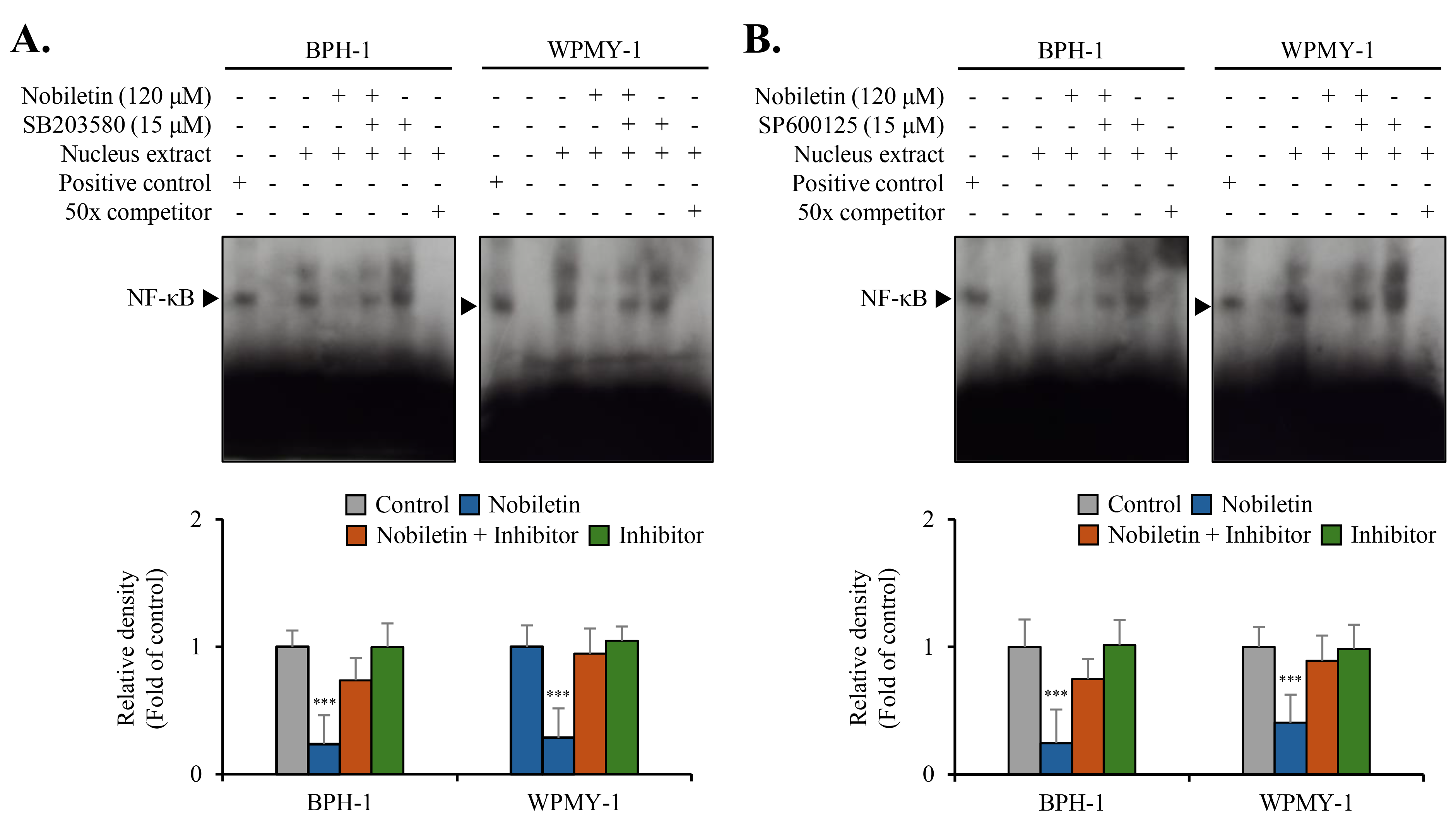

Supplement: Supplementary file 3 [file Image2.tif]

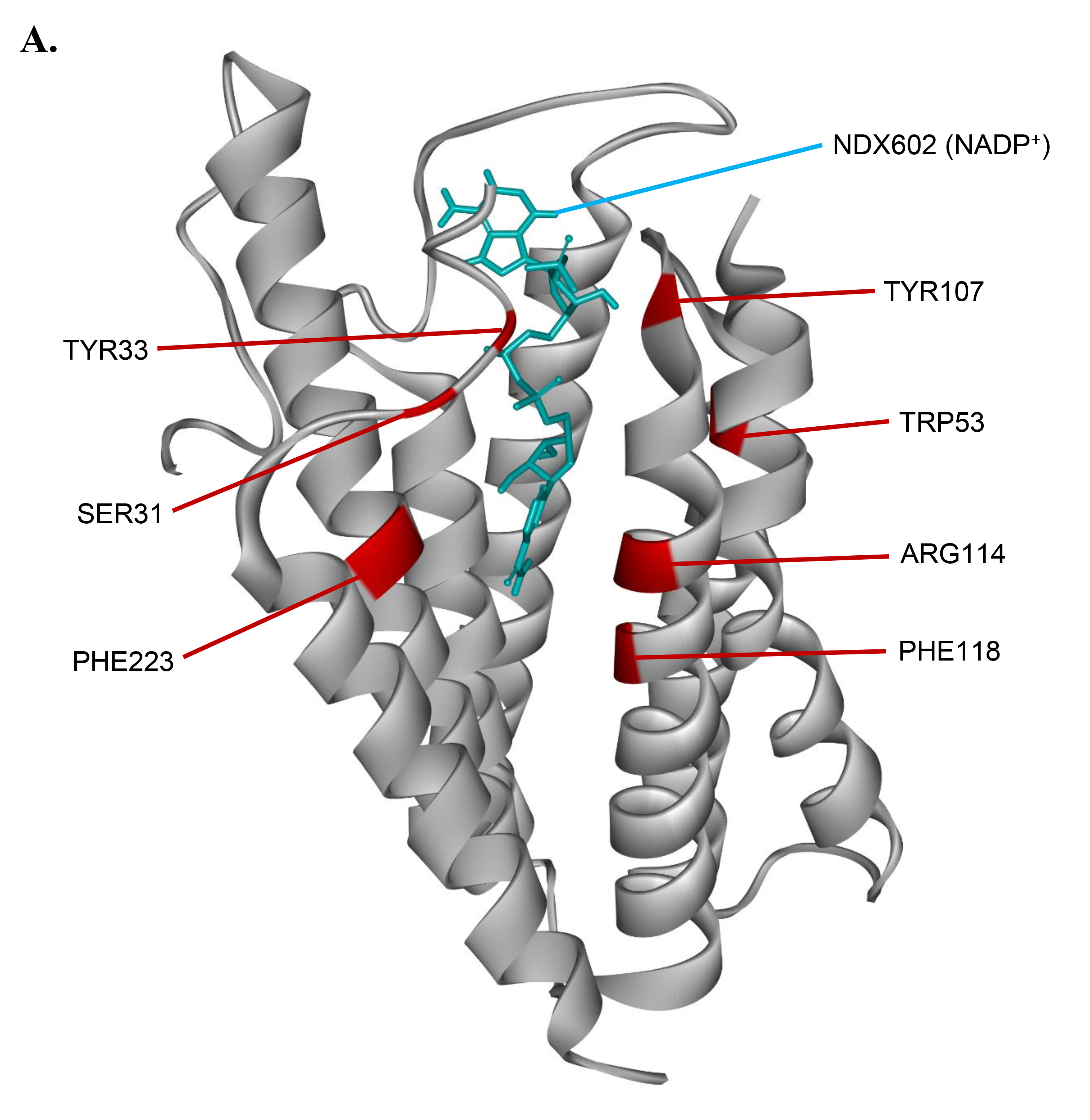

Supplement: Supplementary file 4 [file Image1.tif]
